# Rac-GEF/Rac Signaling and Metastatic Dissemination in Lung Cancer

**DOI:** 10.3389/fcell.2020.00118

**Published:** 2020-02-25

**Authors:** Mariana Cooke, Martin J. Baker, Marcelo G. Kazanietz

**Affiliations:** Department of Systems Pharmacology and Translational Therapeutics, Perelman School of Medicine, University of Pennsylvania, Philadelphia, PA, United States

**Keywords:** Rac, Rac-GEF, lung cancer, adenocarcinoma, tumorigenesis, metastasis

## Abstract

Lung cancer is the leading cause of cancer-related deaths worldwide, with non-small cell lung cancer (NSCLC) representing ∼85% of new diagnoses. The disease is often detected in an advanced metastatic stage, with poor prognosis and clinical outcome. In order to escape from the primary tumor, cancer cells acquire highly motile and invasive phenotypes that involve the dynamic reorganization of the actin cytoskeleton. These processes are tightly regulated by Rac1, a small G-protein that participates in the formation of actin-rich membrane protrusions required for cancer cell motility and for the secretion of extracellular matrix (ECM)-degrading proteases. In this perspective article we focus on the mechanisms leading to aberrant Rac1 signaling in NSCLC progression and metastasis, highlighting the role of Rac Guanine nucleotide Exchange Factors (GEFs). A plausible scenario is that specific Rac-GEFs activate discrete intracellular pools of Rac1, leading to unique functional responses in the context of specific oncogenic drivers, such as mutant EGFR or mutant KRAS. The identification of dysregulated Rac signaling regulators may serve to predict critical biomarkers for metastatic disease in lung cancer patients, ultimately aiding in refining patient prognosis and decision-making in the clinical setting.

## Introduction

Lung cancer causes the most cancer-related deaths worldwide, representing ∼25% of annual cancer fatalities. The disease is often diagnosed in an advance stage when distal and secondary lung metastases are present. Unfortunately, these patients have poor prognosis, with a median survival <1 year. Based on histologic features, lung cancer has been categorized into two main types, non-small cell lung carcinoma (NSCLC) and small cell lung carcinoma, accounting for approximately 85% and 15% of all lung cancers, respectively. The three main histological subtypes of NSCLCs include adenocarcinoma (∼40–50%), squamous cell carcinoma (∼25–30%) and large cell carcinoma (∼10–15%) (Travis et al., 2015). With the emergence of molecular profiling approaches, genomic and epigenomic alterations have been identified in the different lung cancer subtypes. This has provided a thorough understanding of differential oncogenic drivers and deregulated signaling pathways. According to the first comprehensive molecular profiling of lung adenocarcinomas, the most common somatic mutations in lung adenocarcinomas occur in *TP53* (46%), *KRAS* (33%), *KEAP1* (17%), *STK11* (17%) *EGFR* (14%), *NF1* (11%), and *BRAF* (10%), and less frequently in *MET*, *PIK3CA*, *RB1*, *CDKN2A* among other genes (The Cancer Genome Atlas Research Network, 2018). Interestingly, *EGFR* driver mutations occur with the highest frequency in East Asian, females and non-smokers. Conversely, driver *KRAS* mutations are mostly detected in lung adenocarcinomas in life-long smoker patients. Most driver alterations in lung adenocarcinomas, such as mutations in *KRAS* and *EGFR*, *ALK* fusions, and loss of *NF1* function, confer activation of the Ras/Raf/ERK pathway. A different pattern of genetic alterations has been observed in squamous cell carcinomas and small cell lung carcinomas, as expected from the major histological differences in each lung cancer subtype (Hammerman et al., 2012; Peifer et al., 2012; Rudin et al., 2012; Campbell et al., 2016; Inamura, 2017).

Metastasis is a multistep biological-process that includes local invasion by the cancer cell, intravasation, survival in circulation, extravasation, and growth at secondary distal sites. In order to escape from the primary tumor, cancer cells acquire highly motile and invasive traits, partially through the induction of epithelial-to-mesenchymal transition (EMT) and the secretion of proteases responsible for extracellular matrix (ECM) degradation (Lambert et al., 2017). The high migratory capacity of cancer cells involves the dynamic reorganization of the actin cytoskeleton, a process that is tightly regulated by monomeric Rho G-proteins. This family of GTPases encompasses 20 members, with the most prominent corresponding to the Rac, Rho, and Cdc42 subfamilies. In particular, the small G-protein Rac1 plays a key role in the formation of actin-rich projections, such as lamellipodia and ruffles required for cancer cell motility, and invadopodia which leads to ECM degradation through the deposition of proteases (Cook et al., 2014; Casado-Medrano et al., 2018; Lawson and Ridley, 2018). The past two decades have witnessed remarkable advances toward unraveling the roles of Rac1 and related GTPases in oncogenic and metastatic pathways. Particularly for lung cancer, Rac1 has been established as a *bona fide* effector of tyrosine kinase receptors (TKRs) that play prominent roles in disease initiation and progression, including EGFR (Caino et al., 2012; Murillo et al., 2018). Indeed, conditional deletion of the *Rac1* gene in mice delays lung tumor formation driven by mutant Kras^*G*12*D*^ (Kissil et al., 2007). Nonetheless, the intertwined receptor-effector networks leading to Rac1 activation in lung cancer remain ill defined. Hence, deciphering these intricacies and the specific mechanisms behind oncogenesis and metastatic dissemination is required in order to successfully achieve the development of novel therapeutic approaches targeting these modules. This perspective article will frame the highly complex mechanisms controlling Rac1 activation and their prospective clinical value to predict metastatic disease in lung cancer patients.

## Rac1 Cycling Deregulation: It Never Gets Easier, It Just Goes Faster

Similar to most members of the Rho family of guanine-nucleotide binding proteins, Rac1 is a small GTPase (Mw ∼ 21 kDa) that cycles between active GTP-bound and inactive GDP-bound states, and is the most prominently expressed Rac GTPase in lung cancer (Caino et al., 2012). The related Rac3 isoform is also expressed in lung cancer cells (Zhang et al., 2017). The activation of Rac1 is promoted by Guanine nucleotide Exchange Factors (GEFs), proteins responsible for enabling the displacement of bound GDP. Nucleotide exchange then occurs because GTP is in greater excess in the cytosol. Most importantly, this is the key event for the activation of Rac1 downstream signaling. A classical and well-established paradigm of Rac1 signaling is the binding of Rac1-GTP to effectors such as Pak1. Such protein-protein interactions promote changes in actin cytoskeletal dynamics, including the polymerization of actin filaments at the leading edge of the cell to form lamellipodia and ruffles. The inactivation of Rac1 is mediated by GTPase-Activating Proteins (GAPs), which are responsible for accelerating the intrinsic GTP hydrolysis activity. When in the GDP-bound state, Rac1 is sequestered in the cytoplasm and maintained in an “off-state” by Guanine-nucleotide Dissociation Inhibitors (GDIs), which also prevent its unfolding and further degradation (Kazanietz and Caloca, 2017; Bustelo, 2018; Casado-Medrano et al., 2018; Cooke et al., 2019).

While Rac1 cycling between GDP- and GTP-bound states mediated by GEFs and GAPs is essential to elicit biological functions, other regulatory mechanisms also play relevant roles in modulating its cellular activity. For instance, a post-translational lipidation that involves the incorporation of a prenyl group in the C-terminus of Rac1 is fundamental for plasma membrane targeting, therefore facilitating the interaction with GEFs. In addition, Rac1 phosphorylation at Ser71 by Akt has been shown to determine downstream effector specificity and degradation, while Y64 phosphorylation by non-receptor tyrosine-kinases (e.g., FAK, Src) regulates targeting to focal adhesions and GEFs (Chang et al., 2011; Schwarz et al., 2012; Zhao et al., 2013; Cook et al., 2014; Abdrabou and Wang, 2018; Bustelo, 2018). Still, the relevance of these phosphorylation events in the progression of lung cancer or other neoplasms remains to be properly defined, particularly with how these relate to cell motility and invasiveness. Another important Rac1 post-translational modification involves ubiquitination, a central event that determines protein degradation via the proteasome. The E3 ubiquitin ligase responsible for Rac1 degradation, the tumor suppressor HACE1 (HECT Domain and Ankyrin Repeat Containing E3 Ubiquitin Protein Ligase 1), is prominently down-regulated or mutated in various cancers, leading to enhanced Rac1 signaling, that ultimately correlates with disease progression (Zhao et al., 2013; Cook et al., 2014; Li et al., 2019). Analysis of the TCGA-LUAD database^1^ shows HACE1 somatic mutations in 2.1% of lung adenocarcinoma patients, suggesting a potential causal relationship with Rac1 hyperactivation.

*KRAS* driver mutations observed in NSCLC display slowed down GTPase activity, and as a consequence the GDP/GTP ratio shifts toward the GTP-bound active KRAS state. Thus, the cycling motivational phrase “it never gets easier, you just go faster” does not necessarily apply to *KRAS* oncogenic mutations; however, it may well apply to Rac1. Indeed, the mutant Rac1^*P*29*S*^ (highly frequent in melanoma) represents a unique class of mutant in which GTP hydrolysis is not affected, but rather displays elevated GDP/GTP exchange activity (Davis et al., 2013). However, unlike *KRAS*, point mutations in *RAC1* are rare in lung cancer. Rather, lung adenocarcinomas have been reported to up-regulate a spliced variant of *RAC1* with a 19-amino acid in-frame insertion known as Rac1b, particularly in tumors from smokers and patients who showed node positive disease (Stallings-Mann et al., 2012). Unlike Rac1 point mutants, Rac1b displays both impaired GTP hydrolysis and increased GDP/GTP exchange (Melzer et al., 2019). Inducible expression of Rac1b in the lung epithelium of transgenic mice results in tissue architecture features consistent with activation of EMT, with a concomitant increased expression of mesenchymal markers (e.g., vimentin) and down-regulation of epithelial markers (e.g., E-cadherin). Induction of Rac1b expression in lung cancer cells leads to a bypass of KRAS-associated senescence and up-regulates expression of metalloproteases MMP-3 and MMP-9 (Stallings-Mann et al., 2012). Consistent with these findings, Rac1b synergizes with an oncogenic Kras allele to accelerate lung tumor growth (Zhou et al., 2013). Lastly, analysis of databases revealed amplification of the *RAC1* gene in lung adenocarcinomas, ranging from 3 to 5.5% depending on the database (De et al., 2019). Nonetheless, to date there is no evidence for a causal relationship between elevated Rac1 expression levels and lung cancer progression and metastasis. Mechanistically, how Rac1 up-regulation could promote a pro-invasive phenotype without an evident hyperactivated status cannot be easily explained.

## Aberrant Rac-Gef Signaling: Dangerous Liaisons

**FIGURE 1:**
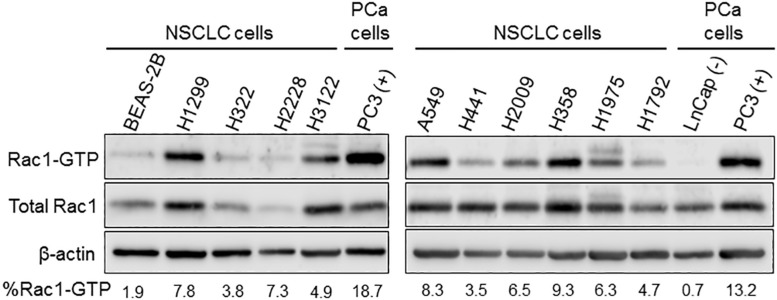
Basal Rac1-GTP levels in NSCLC cells. Cells were serum starved for 16 h, and Rac1-GTP levels were determined using a pull-down assay, as previously described (Caino et al., 2012). As controls, we used a “normal” bronchial epithelial cell line (BEAS-2B). For comparison, we also used prostate cancer cell lines with low basal (LNCaP) and high basal (PC3) Rac-1-GTP levels. Representative Western blots with their corresponding estimation of the active (Rac-GTP) fraction, calculated from densitometric analysis, are shown.

The reported anti-tumor effect of Rac1 and Pak1 inhibitors in lung cancer models and the evidence for enhanced Pak1 activation in lung cancer (Gastonguay et al., 2012; Mortazavi et al., 2015; Wu et al., 2016) are strong indicators of Rac1 hyperactivation. Indeed, a number of NSCLC cancer cells growing in culture display elevated basal levels of Rac1-GTP, providing strong evidence for Rac1 signaling hyperactivation (Figure 1). Considering the low frequency of alterations in Rac1 itself, then other mechanisms should be primarily responsible for the elevated Rac1 activation in lung cancer cells. Most notably, deregulation of the Rac1-GDP/Rac1-GTP cycle may be the consequence of excessive upstream inputs from membrane surface receptors and/or Rac-GEFs, or due to down-regulated GAP function (Cook et al., 2014; Kazanietz and Caloca, 2017; Casado-Medrano et al., 2018). Such changes would shift the cycle toward Rac1-GTP and confer a pro-motile/invasive state.

Despite our understanding of the basis of Rac1 activation, the mechanistic foundation of deregulated Rac1 signaling in lung cancer remains poorly understood. A downside is actually the large number of upstream Rac1 activators and the limited understanding of their expression and regulation in the different lung cancer subtypes. There are more than 80 GEFs in the genome capable of promoting GDP/GTP exchange activity on Rho GTPases, with more than half of them acting on Rac isoforms. Structurally, Rac-GEFs can be divided into two classes: Dbl-like and DOCK GEFs. Dbl Rac-GEFs comprise at least 30 members, in all cases having a characteristic Dbl homology (DH) catalytic domain responsible for promoting GDP/GTP exchange. They also commonly have one or more PH domains capable of binding phosphoinositides, which have been shown to regulate the catalytic activity and localization of some GEFs. Dbl-like Rac GEFs could be structurally as simple as a DH-PH tandem (e.g., ARHGEF39) or possess multiple additional protein-protein interaction domains (e.g., Vav, Tiam, and P-Rex GEFs) (Cook et al., 2014; Gadea and Blangy, 2014; Laurin and Cote, 2014). A level of complexity in Rac-GEF biology is presented by their ability to act on other Rho G-proteins, namely RhoA and/or Cdc42, thus providing mechanisms of signaling divergence for cytoskeletal rearrangements (Cook et al., 2014; Kazanietz and Caloca, 2017; Cooke et al., 2019). Post-translational modifications may also regulate GEF activity and/or localization, including phosphorylation by tyrosine-kinases (e.g., on Vav1) and serine/threonine kinases (e.g., on FARP2) (Bustelo, 2001; Elbediwy et al., 2019). DOCK GEF family members (DOCK1 to 11) contain a catalytic DOCK-homology region (DHR2) and a targeting domain (DHR1). DOCK1-5 act in partnership with ELMO adaptor proteins and display remarkable Rac specificity, whereas DOCK6-11 do not dimerize with ELMO adaptors and also display Cdc42 exchange activity (Cook et al., 2014; Gadea and Blangy, 2014; Laurin and Cote, 2014).

**FIGURE 2:**
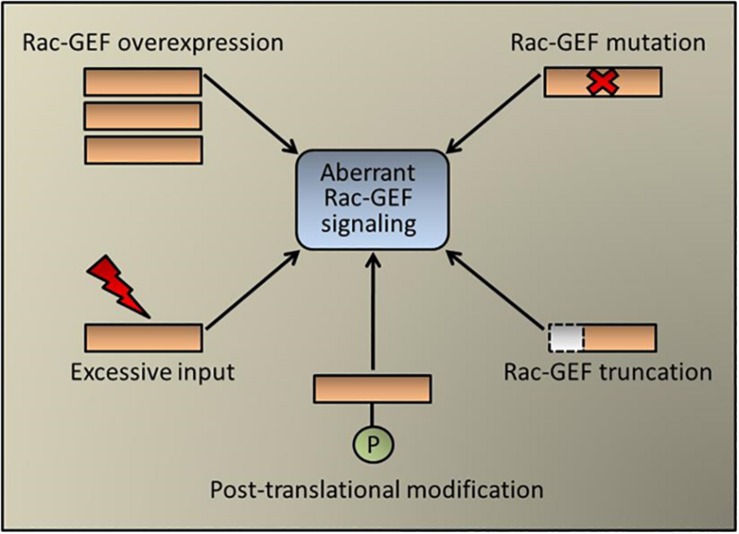
Aberrant Rac-GEF signaling in cancer. Schematic representation of pluricausal events leading to Rac-GEF dysregulation and abnormal Rac1 activation. These mechanisms include Rac-GEF overexpression (e.g., P-Rex1 in breast cancer), point mutations (e.g., SOS1 and Vav1 in lung cancer), protein truncation (e.g., P-Rex2 in melanoma), post-translational modification (e.g., FARP2 in colorectal cancer, which acts also on Cdc42) or an excessive upstream input (e.g., EGFR mutation in lung cancer).

Conceptually, aberrant expression of a Rac-GEF in cancer cells could redirect receptor-mediated signaling toward strengthening Rac1 activation and cell motility, as established for example for the ErbB-receptor activated Rac-GEF P-Rex1 in luminal breast cancer (Sosa et al., 2010). Although the expression of Rac-GEFs in the different lung cancer types has been poorly investigated, it is expected from database analysis that differences in expression between tumor and normal specimens may exist, which would need to be confirmed via histochemical and molecular means. Interestingly, recent exome sequencing analysis identified mutations in *VAV1* and the dual Ras/Rac GEF SOS1 in lung adenocarcinomas. *SOS1* mutations occur at ∼1% frequency in human lung adenocarcinomas, particularly in “oncogene-negative” cases. A number of these SOS1 mutants display oncogenic transforming activity that depends on both Ras- and Rac-GEF activities. Mutations in *VAV1* identified in human lung adenocarcinoma also confer oncogenic activity that can be attributed to their enhanced Rho/Rac activity (Campbell et al., 2016; Shalom et al., 2018; Cai et al., 2019). As Rac-GEFs exist in an auto-inhibited state (He et al., 2013; Cook et al., 2014; Bandekar et al., 2019), it is also possible that truncating deletions may confer constitutive exchange activity as has been described in melanoma for P-Rex2 (Lissanu Deribe et al., 2016). However, this scenario has not yet been explored in the different lung cancer types. Regardless of the absence of recognizable driver mutations in most lung tumors, Rac-GEFs are well known effectors of EGFR and other TKRs, largely via activation of PI3K (Sosa et al., 2010; Zhu et al., 2015; Murillo et al., 2018). Furthermore, the reliance of KRAS on Rac1 in lung cancer development also predicts Rac-GEFs as KRAS effectors, also potentially via a PI3K-dependent mechanism (Murillo et al., 2018). Not surprisingly, genetic deletions or pharmacological inhibition of Rac-GEFs in mouse models greatly impaired metastatic responses (Strumane et al., 2009; Lindsay et al., 2011; Tajiri et al., 2017). Moreover, a pro-metastatic role for DOCK4, which is induced by TGF-β in lung adenocarcinoma cells, unveiled an unexpected link between EMT, Rac1 activation, and metastatic dissemination (Prakash et al., 2019). A scheme depicting the possible mechanisms of aberrant Rac-GEF signaling is shown in Figure 2.

## Location Is the Key: Why Do Cancer Cells Need Multiple Rac-Gefs to Activate Rac1?

The assortment of Dbl-like and DOCK Rac-GEFs expressed in any given cell type, and their sophisticated mechanisms of regulation, strongly imply that Rac1 is regulating multiple cellular functions in different contexts. It is conceivable that Rac-GEFs have both overlapping and non-overlapping roles, which may include functions unrelated to nucleotide exchange activity. Notably, Rac1 controls a number of cellular functions beyond actin cytoskeleton reorganization, including the control of gene expression, generation of reactive oxygen species and ribosomal biogenesis (Hill et al., 1995; Bosco et al., 2009; Justilien et al., 2017; Abdrabou and Wang, 2018). Such specialized roles may reflect selective activation of different intracellular pools of Rac1, which would depend on Rac-GEFs localized at discrete locations. Crucially, Rac1 can be expressed and activated in a range of intracellular compartments, including the nucleus, endosomes and mitochondria, and a similar scenario has been described for Rac-GEFs (Justilien et al., 2017; Abdrabou and Wang, 2018; Pan et al., 2018; Phuyal and Farhan, 2019). One interesting example is the activation of rRNA synthesis and tumorigenesis in the nucleolus of lung cancer cells by the GEF Ect2, thus implicating Rac1 in ribosomal biogenesis (Justilien et al., 2017), a process associated with tumor growth, EMT and metastasis (Prakash et al., 2019). As indicated above, the multidomain nature of these exchange factors is key for determining their subcellular localization via specific mechanisms in each case. The recent identification of Rac-GAP-mediated nuclear Rac1 inactivation in lung cancer cells also supports the compartmentalization of the Rac-GDP/Rac-GTP cycle (Casado-Medrano et al., 2020).

Rac1 has been widely implicated in the control of proliferation, survival and metabolism, cellular functions that are largely controlled by oncogenic signaling. Rac1 and its effectors can activate mitogenic pathways, including the MAPK cascades, leading to changes in cell cycle progression (Olson et al., 1995; Yang et al., 2008). Rac1 has been consistently involved in the activation of pro-survival pathways in cancer cells, such as NF-κB, thereby counterbalancing the apoptotic responses triggered by chemotherapeutic agents and radiation therapy (Friedland et al., 2007; Han et al., 2013; Wang et al., 2013, 2016; Hein et al., 2016). Regarding cellular metabolism, Rac1 has been widely associated with macropinocytosis, a mechanism of endocytic uptake of extracellular fluid that assists in meeting the increased demand of nutrients required for cancer cells to proliferation (Erami et al., 2017; Ramirez et al., 2019). In NSCLC cells, PI3K/Rac1/Pak1-driven macropinocytosis and engulfing of extracellular proteins represents an adaptive metabolic pathway for surviving in states of glucose deprivation (Hodakoski et al., 2019). A recent study established DOCK1 as an important regulator of macropinocytosis in lung cancer cells, and interestingly, a pharmacological inhibitor of DOCK1 displayed significant anti-invasive and anti-metastatic properties (Tajiri et al., 2017). Rac1 function specialization depending on its own compartmentalization and/or localized Rac-GEF activation may therefore be critical for the cancer cell to adapt to the harsh tumor environment in the primary tumor and metastatic sites. Based on this premise, it is conceivable that the acquired transforming and metastatic capacities of lung cancer cells must depend on multiple rather than single Rac-GEFs. In this scenario, other members of the family may not compensate specific cellular functions controlled by an individual Rac-GEF.

## Final Remarks

**FIGURE 3:**
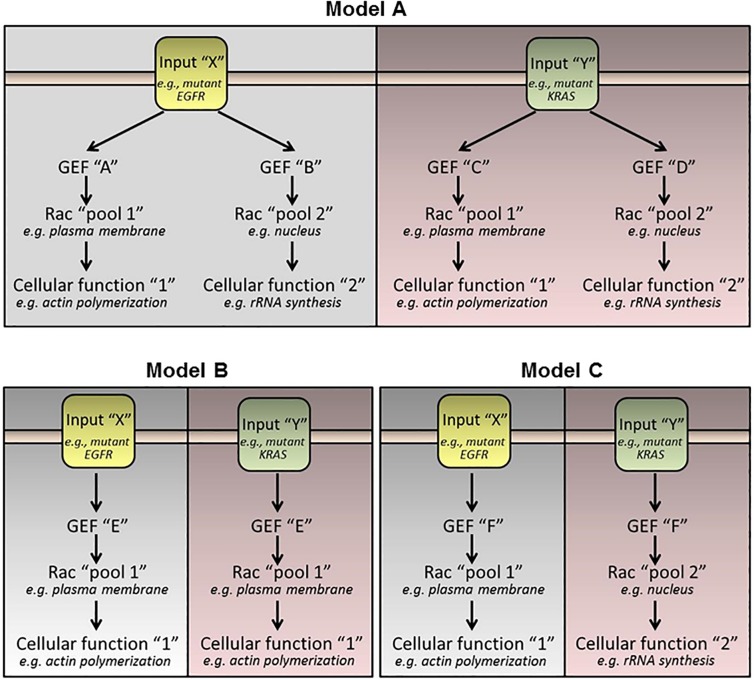
Hypothetical models of Rac-GEF activation by oncogenic stimuli. **(Model A)** Each oncogenic driver activates specific Rac-GEFs. Each Rac-GEF activates a distinctive intracellular pool of Rac1 to promote a specific response. **(Model B)** A Rac-GEF activates a specific pool of Rac1 regardless of the oncogenic driver. **(Model C)** A Rac-GEF activates discrete intracellular pools of Rac1 depending on the oncogenic input.

Rac1 plays fundamental roles in the control of lung cancer development, progression and metastatic dissemination. The convoluted network of receptor-activated Rac-GEFs illustrates the complexity of Rac1-mediated cell biological functions. It remains to be determined whether the expression of pro-metastatic Rac-GEFs differs between the different lung cancer subtypes, and whether their expression and activation changes according to the genetic drivers of the particular tumor subtype. As an example, highly metastatic tumors harboring mutant *EGFR* may utilize a subset of Rac-GEFs distinctive from those driven by *KRAS* mutations, *ALK* fusions, or other oncogenic drivers (Figure 3). These important areas of research deserve to be thoroughly examined, and their mechanistic foundations systematically elucidated.

The many functions that Rac1 plays in the cell and its transient activation provide challenges in targeting it therapeutically. However, an understanding of the GEFs that activate it to drive metastasis in lung cancer, and the mechanism by which these GEFs are facilitating the activation of Rac1, could provide novel opportunities for the development of lung cancer therapeutics. Therefore, evaluating the expression of Rac-GEFs from pleural effusions and other metastatic sites, as well as in circulating tumor cells in the bloodstream from genetically defined lung tumors, will be needed in order to identify novel biomarkers for metastatic disease in lung cancer patients, ultimately aiding in refining diagnosis, prognosis and treatment.

## Data Availability Statement

All datasets generated for this study are included in the article/supplementary material.

## Author Contributions

MC, MB, and MK participated in the designing, writing, and editing of the manuscript, and approved it for publication. MC generated the figures for the article.

## Conflict of Interest

The authors declare that the research was conducted in the absence of any commercial or financial relationships that could be construed as a potential conflict of interest.

## Footnotes

1 https://portal.gdc.cancer.gov/projects/TCGA-LUAD
